# Risk Factors for Cardiovascular Disease, Metabolic Syndrome and Sleepiness in Truck Drivers

**DOI:** 10.5935/abc.20150132

**Published:** 2015-12

**Authors:** Antonio de Padua Mansur, Marcos ABS Rocha, Vilma Leyton, Julio Yashio Takada, Solange Desirée Avakian, Alexandre J Santos, Gisele C Novo, Arledson Lima Nascimento, Daniel Romero Muñoz, Waldo J C Rohlfs

**Affiliations:** 1 Instituto do Coração (InCor) - HC - FMUSP, São Paulo, SP - Brazil; 2 Departamento de Medicina Legal, Ética Médica e Medicina Social e do Trabalho - FMUSP, São Paulo, SP - Brazil; 3 Departamento da Polícia Rodoviária Federal, São Paulo, SP - Brazil

**Keywords:** Cardiovascular Diseases, Risk Factors, Metabolic Syndrome, Hypertension, Obesity, Sleep Stages

## Abstract

**Background:**

Truck driver sleepiness is a primary cause of vehicle accidents. Several causes are associated with sleepiness in truck drivers. Obesity and metabolic syndrome (MetS) are associated with sleep disorders and with primary risk factors for cardiovascular diseases (CVD). We analyzed the relationship between these conditions and prevalence of sleepiness in truck drivers.

**Methods:**

We analyzed the major risk factors for CVD, anthropometric data and sleep disorders in 2228 male truck drivers from 148 road stops made by the Federal Highway Police from 2006 to 2011. Alcohol consumption, illicit drugs and overtime working hours were also analyzed. Sleepiness was assessed using the Epworth Sleepiness Scale.

**Results:**

Mean age was 43.1 ± 10.8 years. From 2006 to 2011, an increase in neck (p = 0.011) and abdominal circumference (p < 0.001), total cholesterol (p < 0.001), triglyceride plasma levels (p = 0.014), and sleepiness was observed (p < 0.001). In addition, a reduction in hypertension (39.6% to 25.9%, p < 0.001), alcohol consumption (32% to 23%, p = 0.033) and overtime hours (52.2% to 42.8%, p < 0.001) was found. Linear regression analysis showed that sleepiness correlated closely with body mass index (β = 0.19, Raj2 = 0.659, p = 0.031), abdominal circumference (β = 0.24, Raj2 = 0.826, p = 0.021), hypertension (β = -0.62, Raj2 = 0.901, p = 0.002), and triglycerides (β = 0.34, Raj2 = 0.936, p = 0.022). Linear multiple regression indicated that hypertension (p = 0.008) and abdominal circumference (p = 0.025) are independent variables for sleepiness.

**Conclusions:**

Increased prevalence of sleepiness was associated with major components of the MetS.

## Introduction

Traffic accidents are an important external cause of death associated with significant social costs. In 2010, Brazil had 320,000 road accidents, of which 35.1% occurred in the Southeastern region and 20.4%, in São Paulo State^1^. Cargo vehicles accounted for over 30% of those accidents, although only 9% of the national vehicle fleet is composed of cargo vehicles^2^.

Several variables have been shown to associate with car accidents^3-6^. Connor et al^3^ have shown that drinking alcohol before driving was responsible for approximately 30% of car crash injuries.^3^ A recent meta-analysis has shown nearly 3 times more vehicle crash risk associated with marijuana^4^. Stutts et al^5^ in a population-based case-control study have reported that drivers in sleep-related crashes were more likely to work multiple jobs, night shifts, or other unusual work schedules. Overtime work, a night shift, unusual work schedule, and ≥ 60 hours per week were associated with sleep-related crashes^5^.

Smolensky et al^6^ reviewed the potential contribution of several prevalent medical conditions on sleep disorders and on traffic crash risk^6^. Obesity and metabolic syndrome (MetS) are prevalent among truck drivers^7,8^ and relate to poor dietary habits and reduced physical activity.

Obesity was associated with some critical security events. Obese truck drivers of heavy commercial vehicles had a 55% higher risk of crash as compared with those with normal weight^9^. Obese truck drivers had higher prevalence of fatigue and risk of involvement in vehicle accidents^10^. Obese drivers involved in vehicle accidents also have higher mortality rate as compared with non-obese drivers^11^.

Obesity and MetS are closely related conditions. MetS is characterized by abdominal obesity, hypertension and metabolic blood alterations, in particular increased blood glucose levels and worsening lipid profiles^12^. Obesity and MetS are strongly associated with obstructive sleep apnea (OSA)^13^, which is an important cause of excessive daytime sleepiness in truck drivers and an important factor associated with accidents^14,15^.

This study analyzed the associations of cardiovascular risk factors, alcohol, and illicit drugs with sleepiness and vehicle accidents.

## Methods

This is a cross-sectional survey on the major cardiovascular risk factors and sleep disorders in 2228 male truck drivers from 148 road stops made by the Federal Highway Police from 2006 to 2011 during the “Commands of Health” program directed to the health of truck drivers carried out once a year on a specific day.

The program is conducted throughout the national territory. Truck drivers were invited to participate and accepting demographic and laboratory data were collected. The response rate was almost 100%. Rare drivers (< 0.5%) refused to participate in the program. All drivers in the study were individual cases, and the likelihood of including the same driver twice in the study was zero.

The interview was conducted and anthropometric data collected by students of nursing or other professions related to human health under the supervision of graduate nurses by using a standard questionnaire. Point-of-care testing was used to analyze the serum levels of glucose, triglycerides and total cholesterol. The demographic data analyzed included personal (age, sex, marital status, ethnicity, educational level, socioeconomic class, neck and abdominal circumferences, and body fat) and occupational information (type of employment, length of daily working hours, driving hours, and sleepiness), as well as self-reported drug use. Amphetamines, marijuana, cocaine and benzodiazepines were the drugs questioned to drivers.

The following cardiovascular risk factors were assessed: smoking, dyslipidemia, diabetes, hypertension, sedentary lifestyle and obesity. The percentage of body fat was calculated using the formula: % of body fat = 495/(1.0324-0.19077(log(waist-neck))+0.15456(log(height)))-450 (log10)^16^. Neck, abdominal, and waist circumferences above normal limits were defined as values ≥ 40 cm, ≥ 102 cm, and ≥ 109 cm, respectively^17^. The obesity risk factor was defined according to body mass index (BMI) (kg/m^2^), using the following scale: normal (BMI ≥ 18.5 to < 25), overweight (BMI ≥ 25 to < 30) and obese (BMI ≥ 30)^18^. Smokers were classified as current versus non-current smokers. Hypertension was diagnosed when systolic blood pressure > 140 mmHg, diastolic blood pressure > 90 mmHg, or if antihypertensive medication was being used^19^. Dyslipidemia was diagnosed in individuals with total cholesterol ≥ 240 mg/dL, triglycerides ≥ 200 mg/dL, low-density lipoprotein (LDL) cholesterol ≥ 130 mg/dL or in individuals using lipid-lowering medications^20^. Diabetes was diagnosed in individuals with fasting glucose ≥ 126 mg/dL or casual plasma glucose ≥ 200 mg/dL as well as in individuals receiving hypoglycemic medications^21^. Sedentary lifestyle was diagnosed qualitatively by self-reported absence or presence of any additional leisure physical activity unrelated to regular working hours.

Sleepiness was assessed using the Epworth Sleepiness Scale with a cutoff score > 10^22^.

The Ethics Committee of the University of São Paulo Medical School approved this study (research protocol n°539/13).

### Statistical analysis

Comparison of percentages and linear regression analysis were used for the statistical analysis of each variable (BMI, diabetes, hypertension, overtime, illicit drugs, alcohol, smoking, hypercholesterolemia, sleepiness, waist circumference, body fat, and triglycerides). All variables were dichotomized and analyzed as a percentage of the presence of the altered variable for each year. Using sleepiness as the dependent variable, linear multivariate regression analyses were performed using diabetes, illicit drug and alcohol use, hypercholesterolemia, hypertriglyceridemia, and abdominal circumference as independent variables. The same analysis was made for vehicle accidents as the dependent variable and diabetes, illicit drug and alcohol use, hypercholesterolemia, hypertriglyceridemia, sleepiness, and abdominal circumference as independent variables. The significance level adopted for the statistical tests was 5% (p < 0.05). The statistical analyses were performed using the SAS program for Windows (Statistical Analysis System version 9.2, SAS Institute Inc., 1989-1996, Cary, NC, USA).

## Results

**Figure 1:**
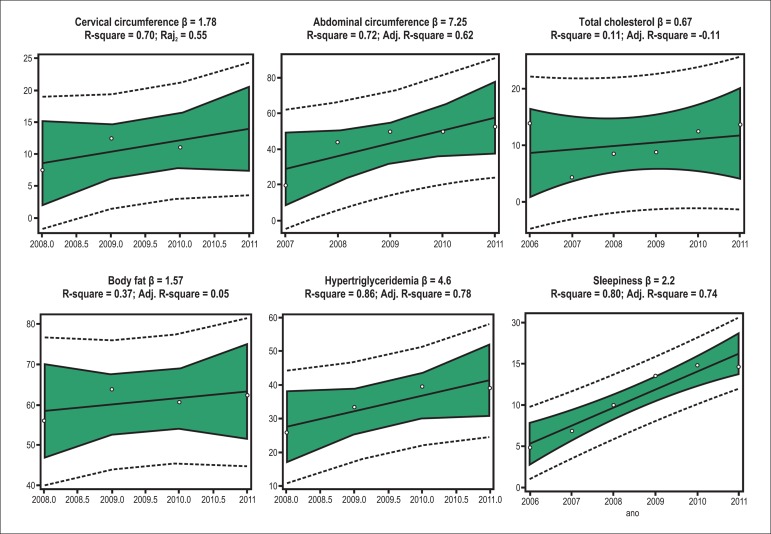
Linear regression results of the variables from 2006/2007 to 2011.

The clinical and laboratory data of 2228 truck drivers from road stops conducted by the Federal Highway Police from 2006 to 2011 are shown in Table 1. The mean age was 43.1 ± 10.8 years. From 2006 to 2011, an increase in neck (7.5% to 13.9%, p = 0.011) and abdominal circumferences (19.8% to 52.8%, p < 0.001), total cholesterol (4.4% to 13.7%, p < 0.001), triglyceride plasma levels (25.8% to 39.1%, p = 0.014), and sleepiness (4.9% to 14.7%, p < 0.001) was observed (Figure 1). In addition, a reduction in hypertension (39.6% to 25.9%, p < 0.001), alcohol consumption (32% to 23%, p = 0.033) and overtime hours worked (52.2% to 42.8%, p < 0.001) was also observed. The data obtained regarding body fat (56.1% to 62.4%, p = 0.395), smoking (20.3% to 17.7%, p = 0.192), hyperglycemia (14.9% to 11%, p = 0.267), and illicit drug use (5.5% to 8.1%, p = 0.127) were similar to the data analyzed from previous years. The linear regression analysis showed that sleepiness was closely correlated with BMI (β = 0.19, Raj2 = 0.659, p = 0.031), abdominal circumference (β = 0.24, Raj2 = 0.826, p = 0.021), hypertension (β = -0.62, Raj2 = 0.901, p = 0.002), triglycerides (β = 0.34, Raj2 = 0.936, p = 0.022). Vehicle accidents showed correlation with only BMI (β = 0.21, Raj2 = 0.807, p = 0.024). Linear multiple regression indicated that hypertension (p = 0.008) and abdominal circumference (p = 0.025) are independent variables for sleepiness, and no independent variable was found for vehicle accidents.

**Table 1:** Clinical and laboratory data of truck drivers from road stops conducted by the Federal Highway Police from 2006 to 2011

**Year**	**2011**	**2010**	**2009**	**2008**	**2007**	**2006**
Number of police road stops	26	26	26	26	24	20
Median number of drivers	425	294.5	300	323.1	236.9	648.1
Body mass index (> 25 Kg/m^2^)(%)	64.5	63.8	64.9	65.5	55.2	58.5
Neck circumference (≥ 40 cm)(%)	13.9	11.1	12.5	7.5	13.9	11.1
Abdominal circumference (≥ 102 cm) (%)	52.8	50.3	49.8	43.8	19.8	52.8
Diabetes (%)	11	11.9	10.1	11.8	14.9	14.4
Hypertension (%)	25.9	24	27.7	36.3	39.6	36.3*
Overtime hours (> 8h) (%)	42.8	43.4	20.9	28.7	52.2	49.9*
Illicit drug use (% yes)	8.1	8.5	5.8	4.8	5.5	4.1
Alcohol (% yes)	23	30.9	24.2	30.3	32	36.6*
Smoking (% yes)	17.7	19.6	18.4	20.5	20.3	19.1
Hypercholesterolemia (%)	13.7	12.5	8.8	8.5	13.9	4.4*
Sleepiness (%)	14.7	14.8	13.6	10	6.9	4.9*
Waist circumference (> 109 cm) (%)	52.8	50.3	49.8	43.8	19.8	NA
Body fat (%)	62.4	60.6	63.8	56.1	NA	NA
Hypertriglyceridemia (%)	39.1	39.6	33.4	25.8*	NA	NA
Cargo vehicles accidents (%)	15.1	16.1	16.6	17.4	12.9	NA

NA: Not applicable.

## Discussion

Our study showed that increased sleepiness was associated with the major components of the MetS. Increased abdominal circumference and hypertension were independently associated with sleepiness. It is well known that our population is becoming increasingly obese^23^, and based on data from our study we observed the same trend in truck drivers. Weight gain is often associated with some degree of hypertension, diabetes, dyslipidemia, and, thus, MetS.

Recently, Hirata et al^24^ found an increased prevalence of hypertension, obesity, hyperlipidemia, and hyperglycemia in bus drivers. These findings were related to lifestyle, such as poor dietary habits and low physical activity^25^. Sleepiness in these subjects may be related to some factors, such as overtime work, poor sleep quality, illicit drug and alcohol intake and obesity. Overtime work accounted for approximately 22% of road accidents and associated with higher mortality rate as compared with other causes^26^. Nevertheless, in our study, overtime work remained constant during the study period.

Alcohol intake and illicit drugs are known to be two important factors associated with sleepiness. We observed a reduction in the percentage of alcohol consumption in our truck drivers and the percentage of illicit drug use remained unchanged during the period analyzed suggesting minor, if any, influence of these variables on the expressive increase of sleepiness in our study. Poor sleep quality and obesity are closely related.

It is well known that drowsiness results from OSA, an important factor in this obese population and in subjects with MetS^27,28^.

Moreno et al^29^ used the Berlin Questionnaire to show that smoking and drug use are independent variables associated with increased risk for OSA in our truck driver population. A low risk for OSA was associated with some degree of exercise.

Another study on Brazilian truck drivers indicated that less than 8 hours of daily sleep, age > 40 years, glucose levels > 200 mg/dL, cholesterol levels > 240 mg/dL, snoring, and hypertension are independent factors associated with obesity^29^. Some of these factors are components of the MetS. Xie et al^30^ also showed that a BMI ≥ 30, hypertension, and diabetes are independently associated with OSA in commercial motor vehicle drivers. In our study, central obesity and hypertension are associated with sleepiness. These changes are likely related to the poor dietary habits, due to the high-calorie meals consumed by truck drivers at highway restaurants, and the lack of physical activity, consequent to overtime work. Nevertheless, the number of car accidents did not change during the study period. This finding may be the result of some improvements to the infrastructure and logistics of the transport system^31^. However, improvements to truck drivers’ health may have an important impact on cargo vehicle accidents.

A recent health survey of U.S. long-haul truck drivers showed that 83.4% were overweight/obese, 57.9% experienced sleep disturbances, and approximately 40% reported cardiovascular disease concerns^32^. An additional study of long-haul truck drivers indicated increased cardiovascular disease mortality in those drivers younger than 55 years^33^. This is a key age group in our truck driver population. The incidence of cardiovascular disease in men has significantly increased from that age onward. Therefore, this is a key group for implementing preventive interventions.

The main limitation of this ecological study was that we performed the statistical analysis based on a percent of the yearly grouped variable instead of the individual subject data. Because we used the percent data indicative of the presence or absence of a particular variable, we could not quantify the intensity of each variable. Other study limitations were: accuracy of point-of-care testing used to analyze the serum levels of glucose, triglycerides and total cholesterol. A common problem in studies with this design is the reverse causality that can mask the effects of some investigated associations. Residual confounding and selection bias are variables that may have influenced our results.

## Conclusion

Increased sleepiness was associated with the major components of the MetS. The implementation of preventive measures, such as improvement in eating habits and physical activity, regular working times, and better working conditions, may reduce cardiovascular disease in this population. Lifestyle changes and cardiovascular risk factor control may reduce sleepiness and consequently decrease cargo vehicle accidents.
